# Dihydroartemisinin Suppresses Hepatocellular Carcinoma Progression by Acting on KIF11 with PI3K/Akt Modulation

**DOI:** 10.3390/cancers18101530

**Published:** 2026-05-09

**Authors:** Aina Xiao, Yu’E Liu, Wenjia Guo

**Affiliations:** 1 Department of Blood Transfusion, People’s Hospital of Ningxia Hui Autonomous Region, Ningxia Medical University, Yinchuan 750002, China; 2 Department of Hematology/Oncology, Boston Children’s Hospital, Harvard Medical School, Boston, MA 02115, USA; 3 Department of Laboratory Medicine, Shanghai East Hospital, School of Medicine, Tongji University, Shanghai 200092, China

**Keywords:** dihydroartemisinin, hepatocellular carcinoma, KIF11, PI3K/Akt signaling, apoptosis

## Abstract

Background/Objectives: Hepatocellular carcinoma (HCC) remains a leading cause of cancer-related mortality worldwide, with limited effective therapies. Dihydroartemisinin (DHA), a derivative of artemisinin, exhibits potent antitumor activity, but its molecular mechanisms in HCC are unclear. Here, we identified kinesin family member 11 (KIF11) as a critical effector of DHA. Methods: Bioinformatic analyses revealed that KIF11 is significantly upregulated in HCC and associated with poor prognosis, and gene expression profiling suggested its oncogenic role via the PI3K/Akt pathway. Functional studies demonstrated that DHA inhibits HCC cell proliferation, migration, invasion, and colony formation, while inducing apoptosis. Xenograft models of nude mice were established for validation. Results: DHA downregulated KIF11 and epithelial–mesenchymal transition markers, whereas KIF11 overexpression attenuated DHA’s inhibitory effects; the inhibition of PI3K restored DHA sensitivity in KIF11-overexpressing cells. In vivo, DHA markedly suppressed tumor growth and malignancy in xenograft models, consistent with modulation of KIF11 and EMT-related proteins. Conclusions: DHA exerts antitumor effects in HCC by acting via KIF11 and PI3K/Akt modulation, providing a potential therapeutic strategy.

## Simple Summary

This is the first study to demonstrate that dihydroartemisinin (DHA) suppresses HCC cell proliferation, migration, invasion, and colony formation—and induces apoptosis—by acting via KIF11 and PI3K/Akt pathway modulation in vitro and in vivo. We also systematically evaluated KIF11’s oncogenic role in HCC using bioinformatics.

## 1. Introduction

Hepatocellular carcinoma (HCC) ranks sixth in global incidence and third in mortality worldwide [1]. Globally, there were approximately 860,000 new cases and 750,000 deaths in 2022 [2]. Epidemiological models project a 122% increase in the incidence of liver cancer in the United States between 2016 and 2030, with the number of new cases potentially reaching 1.3 million by 2040 [3,4]. The pathological basis for the prolonged onset of this disease is attributed to the liver’s robust compensatory capacity, resulting in a latent clinical presentation with atypical early symptoms. More concerning is that therapeutic options for advanced liver cancer remain limited and often ineffective, with only 30% of patients responding to newer immune checkpoint inhibitors. Compared to other cancers, survival rate of liver cancer has shown minimal improvement in 1995–2022, and the 5-year survival rate remains below 20% [5].

Dihydroartemisinin (DHA), an FDA-approved antimalarial drug, also exhibits significant potential in anticancer therapy. This artemisinin-derived compound is highly regarded among researchers due to its selective toxicity toward malignant cells, broad antitumor spectrum, multi-targeting capabilities, immune modulation, ability to reverse drug resistance, and capacity to enhance sensitivity to radiotherapy and chemotherapy. Currently, the development of DHA-based formulations remains in the preclinical stage, with phase II and phase III clinical trials pending to validate clinical efficacy [6]. The peroxide bridge enables DHA to release free radicals, which can trigger reactive oxygen species (ROS) or carbon-centered free radical-mediated DNA damage [7,8]. These damaging effects compromise cell integrity, disrupt DNA replication, and arrest the cell cycle. In addition, DHA induces apoptosis in liver cancer cells predominantly during the G2/M phase through multiple pathways [9], including caspase activation [10], metabolism regulation [11], mitochondrial membrane depolarization and cytochrome c release [12]. Moreover, DHA enhances the therapeutic efficacy of anti-PD-1 immunotherapy by inhibiting the highly expressed YAP1 in liver cancer [13]. Regarding current standard-of-care systemic therapies for HCC, DHA combined with cisplatin significantly suppressed murine HCC growth, alleviated cisplatin-induced weight loss and splenic atrophy, and reduced intra-tumoral TGF-β—thereby reversing cisplatin-driven immunosuppression and enhancing therapeutic efficacy [14]. Hou et al. further demonstrated that DHA synergized with sorafenib to potentiate antiproliferative effects in HCC cells [15]. Similarly, DHA enhanced the cytotoxicity of gemcitabine in vitro, reducing HCC cell survival by 1.2-fold compared with gemcitabine monotherapy [16]. Collectively, DHA exerts broad antitumor effects by inhibiting proliferation, inducing apoptosis, and correcting genomic abnormalities.

Nine hub genes were identified using the MCC algorithm in our previous research on DHA targeting liver cancer using network pharmacology [17], with gene symbols CDK1, CCNA2, CCNB1, CCNB2, KIF11, CHEK1, TYMS, AURKA, and TOP2A. Subsequently, molecular docking between DHA and the corresponding proteins was performed using AutoDock software (4.2.6), stating that DHA could stably bind to all nine hub proteins [18]. Functional enrichment analysis revealed that these potential targets are primarily involved in fundamental cellular processes, including cell cycle regulation, mitosis, and DNA repair [19,20]. Among which, KIF11, also known as kinesin spindle protein, Eg5, or kinesin-5, is a member of the kinesin superfamily that plays a critical role in the formation and maintenance of bipolar spindles during eukaryotic mitosis. It possesses microtubule polymerase activity and functions by tracking the plus ends of microtubules to regulate their dynamics. The overexpression of KIF11 can lead to centrosome fragmentation, chromosomal instability, and uncontrolled cell division. The inhibition of KIF11 disrupts mitotic progression, induces cell cycle arrest, and ultimately triggers cell death [21]. Beyond its role in mitosis, KIF11 contributes to intracellular transport in non-dividing cells, including the trafficking of secretory proteins from the Golgi complex to the plasma membrane and the facilitation of translation through physical coupling between ribosomes and microtubules [22,23]. KIF11 is frequently overexpressed in various human cancers [24,25], and both the genetic silencing and pharmacological inhibition of KIF11 have demonstrated significant tumor-suppressive effects in vitro and in vivo [26,27,28]. Therefore, KIF11 represents a promising candidate for targeted cancer therapy and warrants further investigation. KIF11 inhibitors represent a second generation of mitotic inhibitors that specifically target KIF11 to prevent bipolar spindle assembly, thereby blocking mitosis [29,30,31]. Unlike first-generation anti-mitotic drugs such as taxanes and epothilones, which exert effects by directly inhibiting microtubule dynamics and therefore cause severe toxicities including neurological disorders with resistance [28,32], this inhibitor’s microtubule-independent effect reduces reliance on tubulin targeting and is associated with a lower incidence of adverse effects [30,33,34]. Several KIF11-targeting agents, including Ispinesib and SB-743921, are currently undergoing clinical evaluation [31,35]. Moreover, KIF11 has emerged as a key molecular target of various traditional Chinese medicines and bioactive monomers in anticancer research. For instance, cyclovirobuxine D suppresses proliferation, invasion, migration, angiogenesis, and epithelial–mesenchymal transition in non-small cell lung cancer cells by inhibiting the KIF11-CDK1-CDC25C-cyclinB1 signaling axis, resulting in a significant reduction in both the size and weight of NSCLC xenograft tumors in nude mice [36]. Additionally, Jun’s integrative analysis of multiple disease and pharmacological databases suggests that KIF11, along with CCNA2 and CDC25A, may serve as core targets mediating dehydroabietic acid-induced pyroptosis in liver cancer cells, with marked downregulation observed at both the mRNA and protein levels following treatment [37]. Therefore, we hypothesize that DHA exerts antitumor effects on liver cancer—particularly in terms of suppressing proliferation, apoptosis, migration, and invasion—through the modulation of KIF11, and we further investigate the underlying molecular mechanisms and signaling pathways involved.

## 2. Materials and Methods

### 2.1. Bioinformatics Analysis

TIMER 2.0 and Sangerbox (http://sangerbox.com/; accessed on 1 May 2026) were utilized to compare the expression of KIF11 between human tumors and normal tissues [38]. Transcriptome data was extracted from The Cancer Genome Atlas Program (TCGA) and GTEx databases [39,40]. The Gene Expression Profiling Interactive Analysis (GEPIA) online tool was used to explore the differences along the stages [41]. Immuno-histochemical (IHC) images of KIF11 in liver cancer from the Human Protein Atlas (THPA) were downloaded [42]. Receiver operating characteristic curves (ROC) were drawn as indexes of KIF11’s diagnosis performance based on TCGA datasets, and the area under curve (AUC) was calculated. Survival data was collected from TCGA and The University of ALabama at Birmingham CANcer data analysis Portal (UALCAN) [43]. Clinical outcomes, namely overall survival (OS), disease-free interval (DFI), disease-specific survival (DSS) and progression-free interval (PFI), were analyzed using Kaplan–Meier graphs as well as Cox regression with a log-rank test to evaluate the prognostic and predictive value of KIF11.

As for transcriptional datasets from Gene Expression Omnibus (GEO) and International Cancer Genome Consortium (ICGC), differentially expressed genes (DEGs) were determined with |log2FC| > 1 and *p* < 0.05. KEGG pathway was conducted with R (4.2.1). *p* < 0.05 was considered statistically significant.

### 2.2. Molecular Dynamics Stimulation

All-atom dynamic simulations were performed on the small-molecule–KIF11 protein (PDB ID: 3ZCW) complex obtained from molecular docking [17,31,44], using GROMACS 2025.1 [45]. KIF11 protein was parameterized with the CHARMM36m force field via CHARMM-GUI, and DHA was assigned parameters using the MMFF94 force field through SwissParam [46,47,48]. Hydrogen atoms were added using the LEaP module (AmberTools24), and the system was solvated in a truncated octahedral box of TIP3P water molecules extending 10 Å beyond the solute surface. Neutralization was achieved by adding Na^+^/Cl^−^ counterions, followed by generation of topology and parameter files for simulation [49].

The simulation protocol comprised four sequential stages: (i) energy minimization using 5000 steps of steepest descent; (ii) 200 ps of NVT equilibration under constant-volume conditions, with temperature gradually raised from 0 K to 310.15 K at a rate of 1.55 K/ps; (iii) 1 ns of NVT production equilibration at 310.15 K to ensure solvent relaxation and stable density; and (iv) 100 ns of unrestrained NPT production simulation under periodic boundary conditions, maintained at 310.15 K and 1 bar using the Berendsen barostat (during equilibration) and Parrinello–Rahman barostat (during production). Nonbonded interactions were treated with a 12 Å cutoff for van der Waals forces and the Particle Mesh Ewald (PME) method for long-range electrostatics. Bonds involving hydrogen atoms were constrained using the SHAKE algorithm, and temperature was controlled via the velocity-rescaling thermostat (V-rescale) with a coupling time constant of 0.1 ps, replacing the less commonly used Langevin algorithm in standard GROMACS membrane/protein protocols. Integration was performed with a 2 fs time step, and coordinates were saved every 10 ps for trajectory analysis [50,51,52].

### 2.3. Cell Lines and Reagents

The human hepatocyte cell line THLE-2 was kindly provided by Shanghai Jiao Tong University with specialized culture medium (Procell, Wuhan, China, CM-0833). HCC cell line HCCLM3 was cultured in high-glucose DMEM (Servicebio, Wuhan, China, G4515) supplemented with 100 U/mL penicillin (Servicebio, Wuhan, China, G4003), 100 mg/mL streptomycin (Servicebio, Wuhan, China, G4003), and 10% fetal bovine serum (Gibco, Carlsbad, CA, USA, A5256501). The human pleomorphic HCC cell line SNU387 was cultured in RPMI-1640 medium (Servicebio, Wuhan, China, G4535) with 10% fetal bovine serum (FBS, Gibco, USA, A5256501), 100 U/mL penicillin (Servicebio, Wuhan, China, G4003), and 100 mg/mL streptomycin (Servicebio, Wuhan, China, G4003). All cell lines were purchased in April 2023, from Servicebio (STCC10108P, STCC10111P), have not been previously reported as misidentified or contaminated, were free of mycoplasma contamination for the described experiments, and were cultured at 37 °C with 5% CO_2_. DHA, CCK8 reagent, plates and Matrigel gel were the same as in our previously conducted research studies [17]. The apoptosis kit was obtained from Elabscience (Wuhan, China, E-CK-A217). RNA extraction was finished with AG RNAex Pro RNA (Agbio, Changsha, China, AG21101). Quantitative real-time PCR (qRT-PCR) reagent was obtained from Vazyme (Nanjing, China, ChamQ Universal SYBR qPCR Master Mix, Q711). Experiments were performed in triplicates. RIPA lysis buffer, Tris-Gly, PVDF membrane and Hypersensitive Chemiluminescent substrate (BeyoECL Plus Kit) were obtained from Beyotime (Shanghai, China, P0038; P0014A; FFP19; P0018S). Marker as well as SDS-PAGE gel were obtained from Epizyme (Shanghai, China, WJ103; PG221). Antibodies for immunohistochemical (IHC), immunofluorescence (IF) and Western blotting (WB) were obtained for Servicebio (Wuhan, China, Ki-67 as GB111499, TUNEL as G1507, VIM as GB11192, CDH2 as GB11135, CDH1 as GB12083, KIF11 as GB112507, GAPDH as GB11002, and β-Tubulin as GB15140). BioDewax and Clear Solution were obtained from Servicebio (Wuhan, China, G1128-1L).

### 2.4. Construction of KIF11-Expressed SNU387 Cell Line

The plasmids pLV3-CMV-KIF11 (human)-3×FLAG-CopGFP-Puro (Wuhan, Miaoling, China, P52640) and the empty control plasmid pLV3-CMV-MCS-3×FLAG-CopGFP-Puro (Wuhan, Miaoling, China, P40122) were obtained. Lentiviral particle packaging and transduction were performed according to standard protocols (Life-ilab, Shanghai, China). SNU387 cells were infected with the generated lentiviral particles and subsequently subjected to puromycin selection (Beyotime, Shanghai, China, ST551). GFP fluorescence was assessed 72 h post-infection to evaluate transduction efficiency.

### 2.5. Clinical Specimens

Surgical specimens were obtained from Department of Hepatobiliary Surgery of Shanghai East Hospital. In total, 10 HCC samples and para-carcinoma tissue were collected. This study was approved by the Medical Ethics Committee of Shanghai East Hospital (2014166). All patients met CSCO guidelines. All procedures followed the ethical standards of Declaration of Helsinki.

### 2.6. CCK-8 Assay

The procedure was consistent with our previous research studies [17], in which an appropriate number of cells were seeded into 96-well plates and treated with DHA for 24, 48, or 72 h. Following incubation, CCK-8 reagent was prepared and added to each well to detect viable cells. The plates were then incubated at 37 °C for 2–4 h and analyzed using an Enzyme-Linked Immunosorbent Detector (Thermo Scientific, Waltham, MA, USA) to measure absorbance at 450 nm.

### 2.7. Flow Cytometry (FC)

SNU387 and HCCLM3 cells were seeded in six-well plates at a density of 2 × 10^5^ cells per well and allowed to adhere for 8 h prior to treatment with indicated concentrations of DHA (20 µM, 50 µM, and 100 µM) for 24 h. Subsequently, both supernatants and cell pellets were collected, washed three times with PBS, and then stained with Annexin V-APC/PI at 4 °C in the dark for 20 min.

### 2.8. Transwell Invasion and Migration

The procedure was conducted in accordance with previous studies [17]. Briefly, HCC cells were suspended in serum-free medium for 12 h, followed by the addition of 200 µL of medium containing 4 × 10^4^ cells in 2% FBS to the upper chamber of a Transwell insert, supplemented with DHA at final concentrations of 20 µM, 50 µM, and 100 µM. The control group received medium with 2% FBS only. The lower chamber was filled with 600 µL of medium supplemented with 10% FBS. After 24 h of incubation, the Transwell inserts were removed, washed, fixed, and stained with crystal violet. Migrated cells on the lower surface of the membrane were then imaged and quantified. The invasion assay followed an identical protocol, except that the Transwell chambers were pre-coated with Matrigel.

### 2.9. Wound Healing

The detailed experimental procedure has been described in our previous study [17]. A wound was created by scratching, and HCC cells were seeded and allowed to form a confluent monolayer before being treated with medium containing 2% FBS and DHA at concentrations of 20 μM, 50 μM, and 100 μM for 24 h. Subsequently, the migration area was analyzed.

### 2.10. Colony Formation Assay

A total of 500 cells per well were seeded into sterile six-well plates and cultured at 37 °C under 5% CO_2_ for 24 h. Subsequently, the medium was replaced with fresh complete medium containing DHA at final concentrations of 20, 50, or 100 µM; untreated cells served as the vehicle control. All plates were maintained at 37 °C under 5% CO_2_ for 10–14 days, with the medium refreshed every 3 days and colony formation monitored daily by phase-contrast microscopy.

Colonies were fixed upon reaching ≥50 cells per cluster—evident both macroscopically and microscopically—by adding 1% paraformaldehyde for 20 min at room temperature, followed by two PBS washes. Cells were then stained with 1 mL of 0.5% crystal violet (in distilled water) per well for 10 min, rinsed thoroughly with distilled water to remove unbound dye, air-dried, and imaged. Colonies containing ≥50 cells were manually counted.

### 2.11. Quantitative Real-Time PCR (qRT-PCR)

Total RNA was extracted from cells using AG RNAex Pro reagent, followed by cDNA synthesis. The primer sequences used for qRT-PCR are stated in Supplementary Table S1. The specificity of all primers was experimentally verified using melt curve analysis. All primers generated a single peak in the melt curve, confirming the specificity of the amplification. All mRNA expression levels were quantified by qRT-PCR using ChamQ Universal SYBR qPCR Master Mix (Vazyme), and relative gene expression was normalized to GAPDH as an internal control.

### 2.12. Western Blotting (WB)

Cellular proteins were extracted using RIPA lysis buffer. Protein samples were then separated by 7.5% SDS-PAGE at 110 V for 50 min and subsequently transferred onto PVDF membranes at 400 mA for 30 min. Membranes were blocked and incubated with specific primary antibodies overnight at 4 °C. The following day, membranes were incubated with corresponding horseradish peroxidase-conjugated secondary antibodies for 1 h, and immunoreactive bands were visualized using the BeyoECL Plus Kit (Beoytime, Shanghai, China).

### 2.13. Immunofluorescence (IF)

Slides were deparaffinized using BioDewax (Servicebio, Wuhan, China) and Clear Solution (Servicebio, Wuhan, China), subjected to antigen retrieval in EDTA buffer, and blocked for nonspecific binding sites with 3% bovine serum albumin. Sections were then incubated with primary antibodies overnight at 4 °C, followed by incubation with secondary antibodies. Finally, slides were counter-stained with DAPI solution at 37 °C for 10 min in the dark. DAPI exhibits an excitation wavelength range of 330–380 nm and an emission peak at 420 nm; CY3 has an excitation range of 510–560 nm and an emission maximum at 590 nm.

### 2.14. Immunohistochemistry (IHC)

Deparaffinization and antigen retrieval were performed as described in Section 2.5. Endogenous peroxidase activity was blocked by incubation with 3% hydrogen peroxide solution, followed by blocking of nonspecific binding sites using 3% bovine serum albumin. The slides were placed horizontally in a humidified chamber and incubated with primary antibody overnight at 4 °C, and then with secondary antibody. Subsequently, DAB chromogenic detection was carried out, and nuclei were counterstained with hematoxylin. Finally, the sections were dehydrated through an ethanol–xylene series and mounted for microscopic evaluation and imaging.

### 2.15. Xenografted Animals

Male nude mice (4 weeks old) were purchased from Gempharmatech Co., Ltd. (Shanghai, China). After one week of adaptive culture, the animals were randomly assigned to experimental groups: SNU387 + saline solution, SNU387 + DHA (50 mg/kg), ov-KIF11 SNU387 + DHA (50 mg/kg), and ov-NC SNU387 + DHA (50 mg/kg), with 1 × 10^6^ cells injected subcutaneously. DHA was administered via intraperitoneal injection for two weeks.

Tumor formation was assessed one week after subcutaneous cell inoculation and drug treatment. Mice were monitored daily for general health and signs of tumor development; tumor-bearing mice were euthanized 14 days post-tumor detection (defined as palpable tumor ≥ 2 mm in diameter). Subcutaneous tumors were excised, photographed, and measured using calipers; the tumor volume was calculated using the formula volume = (length × width^2^)/2 (mm^3^), where L is the longest diameter and W is the perpendicular shortest diameter. Fresh tumor tissues were immediately processed—half were fixed in 4% paraformaldehyde for histological analysis, and the other half were snap-frozen in liquid nitrogen for molecular assays.

All animal procedures were conducted in strict accordance with the 3Rs principles (Replacement, Reduction, Refinement) and approved by the Institutional Animal Care and Use Committee (IACUC) of Shanghai East Hospital.

### 2.16. Statistical Analysis

All statistical analyses were performed Graphpad prism (8.0 version). Numeric data were expressed as the mean ± standard deviation (SD). A Shapiro–Wilk test was performed on continuous data to determine normality. A comparison between two groups was performed by applying an independent sample *t* test or Wilcoxon rank test. For more multiple groups, a One-way ANOVA test was used for comparisons based on normality and corrected using the Bonferroni method. A Spearman correlation test was used to analyze the linear correlation. A logistic as well as log rank test was used for survival-related analysis. Differences between groups were considered significant if *p* < 0.05. The *p*-values are uniformly replaced with the following symbols in all the figures: * *p* < 0.05; ** *p* < 0.01; and *** *p* < 0.001.

## 3. Results

### 3.1. KIF11 Is Overexpressed and Clinically Relevant in HCC

**Figure 1:**
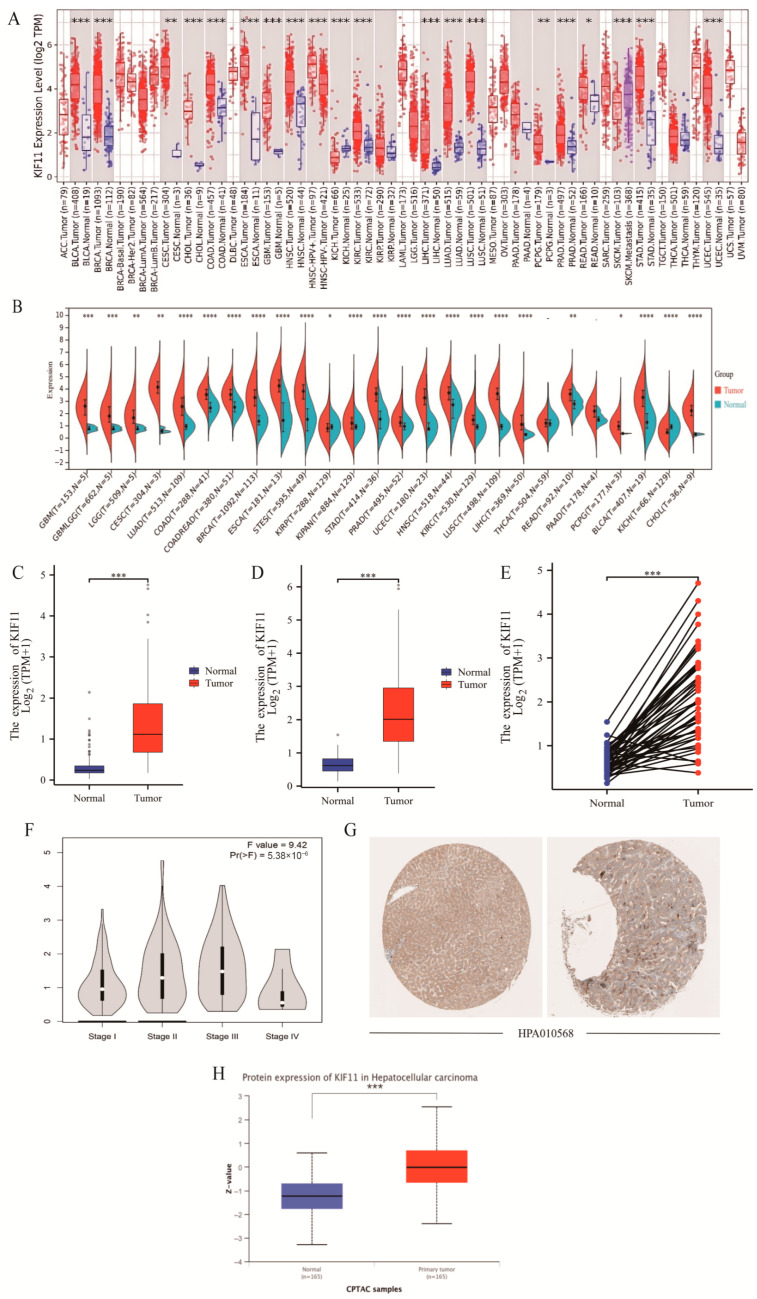
Bioinformatics analysis of KIF11 expression in HCC. (**A**,**B**). Pan-cancer expression analysis of KIF11 using TIMER 2.0 and Sangerbox, using Wilcoxon Rank Sum Test. (**C**) KIF11 expression in TCGA-LIHC and GTEx cohort, using Wilcoxon Rank Sum Test. (**D**) KIF11 expression in TCGA-LIHC cohort, using Wilcoxon Rank Sum Test. (**E**) KIF11 expression in paired tumor and adjacent non-tumor tissues from TCGA-LIHC cohort, using Wilcoxon Rank Sum Test. (**F**) GEPIA of KIF11 expression across pathological stages of HCC, using One-way ANOVA. (**G**) IHC images showing KIF11 protein expression in liver cancer and normal liver tissues from the THPA; (**H**) KIF11 protein expression levels in liver cancer versus normal liver tissues based on GEPIA, using Wilcoxon Rank Sum Test. * *p* < 0.05; ** *p* < 0.01; *** *p* < 0.001; **** *p* < 0.0001.

Pan-cancer analysis using TIMER and Sangerbox databases showed that KIF11 was significantly upregulated in most tumor types (except chromophobe RCC) (Figure 1A,B). Comparisons between 371 HCC samples (TCGA) and 110 normal liver tissues (GTEx) indicated markedly higher KIF11 expression in HCC (*p* < 0.001; Figure 1C–E). Stage-wise analysis via GEPIA revealed significant variation in KIF11 across liver cancer stages (*p* < 0.001), suggesting an association with disease progression (Figure 1F). Protein-level assessment from THPA IHC confirmed stronger KIF11 staining in tumor tissues versus normal liver tissues (Figure 1G), consistent with UALCAN results (*p* < 0.001; Figure 1H).

**Figure 2:**
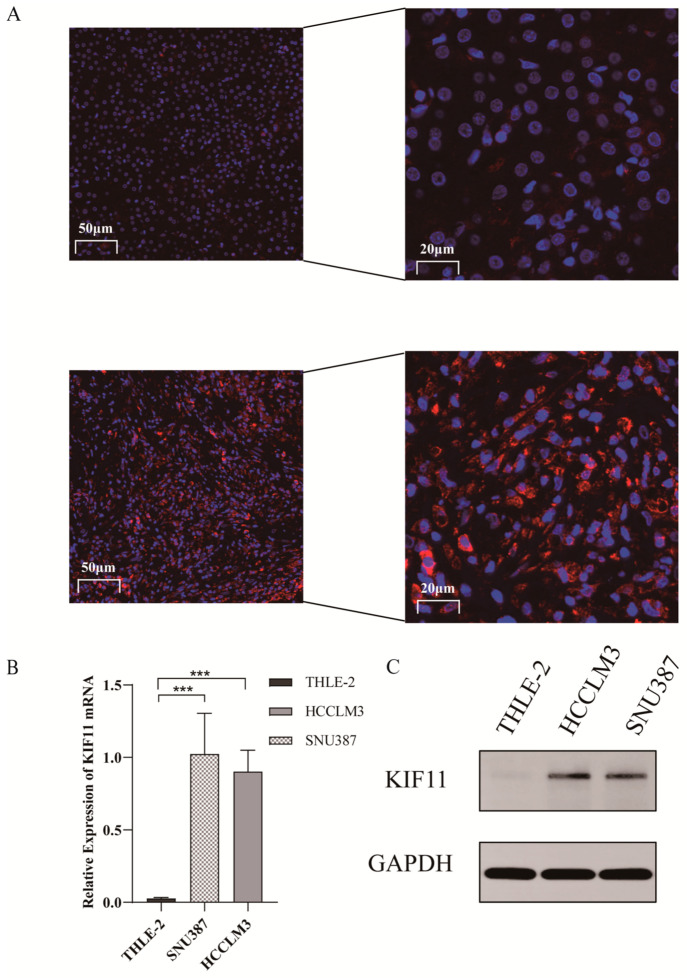
KIF11 is upregulated in HCC. (**A**) IF staining of KIF11 in HCC tissues and paired adjacent non-tumor liver tissues (scale bar = 50 µm, 200×; 20 µm, 630×); (**B**) mRNA expression of KIF11 in the normal human hepatic cell line THLE-2 and HCC cell lines HCCLM3 and SNU387, using One-way ANOVA with Bonferroni test; (**C**) Protein expression of KIF11 in THLE-2, HCCLM3, and SNU387 cells as determined by WB, using One-way ANOVA with Bonferroni test. The uncropped blots and molecular weight markers are shown in Supplementary File S1. *** *p* < 0.001.

Experimental validation supported these bioinformatic observations. Immunofluorescence (IF) of ten paired HCC and adjacent non-tumor sections showed the robust cytoplasmic upregulation of KIF11 in the tumor tissue (Figure 2A). qRT-PCR and Western blotting demonstrated significantly lower KIF11 expression in the normal hepatocyte line THLE-2 compared with HCC lines HCCLM3 and SNU387 (*p* < 0.001; Figure 2B,C).

**Figure 3:**
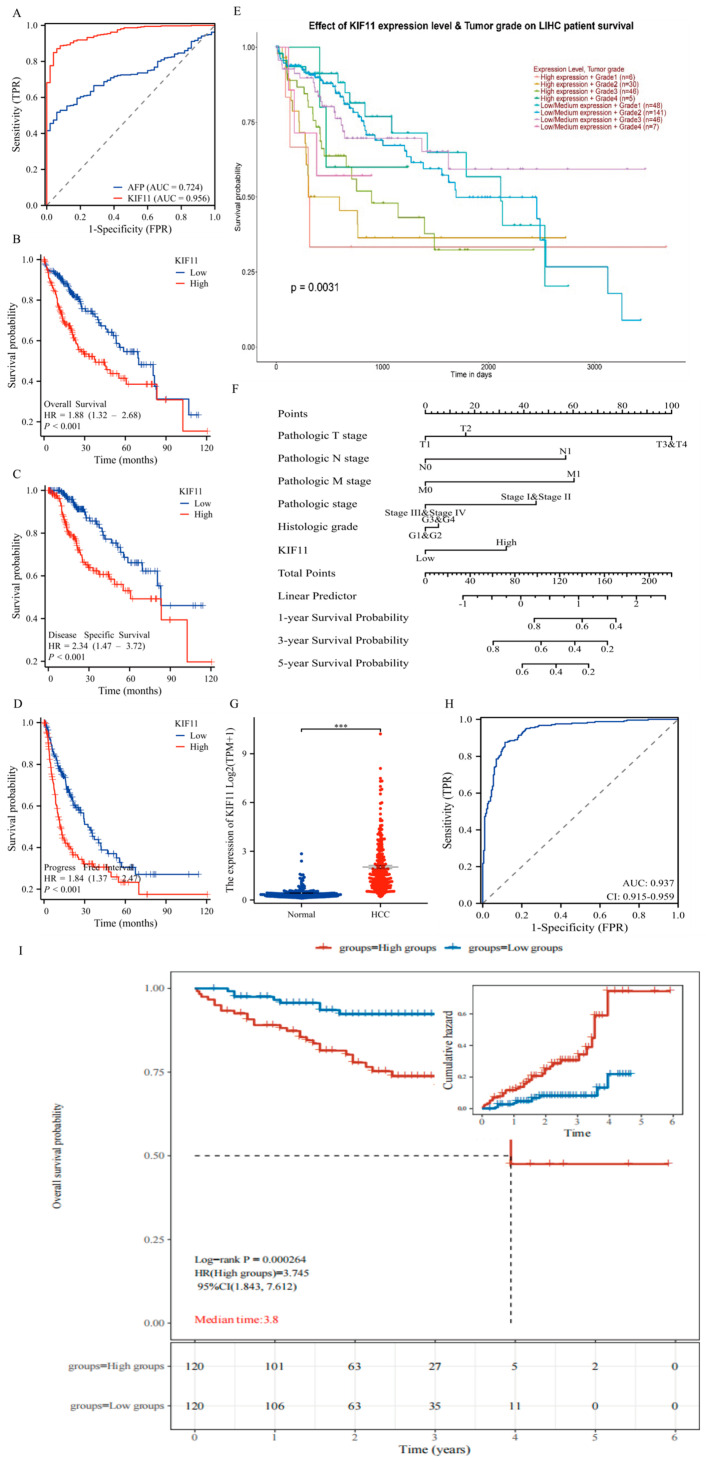
Diagnostic and prognostic significance of KIF11 in HCC. (**A**) ROC curve evaluating the diagnostic accuracy of KIF11 with TCGA-LIHC cohort. (**B**–**D**) Kaplan–Meier survival curves depicting association between KIF11 expression and overall OS, DFS, and PFS in TCGA-LIHC, with log-rank test. (**E**) Kaplan–Meier analysis of KIF11 expression stratified by tumor grade and clinical stage in HCC based on UALCAN, with log-rank test. (**F**) A nomogram integrating KIF11 expression and clinical variables to predict overall survival probability in TCGA-LIHC patients, with Logistic and Cox regression test. (**G**) Differential expression of KIF11 in tumor tissues compared to adjacent non-tumor tissues in the LIRI-JP cohort, using Wilcoxon test. (**H**) ROC curve analysis assessing the diagnostic performance of KIF11. (**I**) Kaplan–Meier survival curves showing the association between KIF11 expression and OS, with log-rank test *p*-values indicated, with log-rank test. *** *p* < 0.001.

Diagnostic and prognostic analyses further established clinical relevance. ROC analysis in the primary TCGA cohort yielded an AUC of 95.6% with a sensitivity of 87.1%, specificity of 94%, and positive predictive value of 99.1%, outperforming AFP (Figure 3A). Kaplan–Meier stratification (median KIF11) showed that high KIF11 expression correlated with worse OS, DFS and PFS (*p* < 0.001; Figure 3B–D). The UALCAN database further confirmed that elevated KIF11 expression is associated with poorer prognosis across different tumor stages and histological grades. A prognostic nomogram incorporating KIF11, histological grade, pathological stage and TNM parameters was constructed and validated. (*p* < 0.05, Figure 3E,F). External validation in the ICGC LIRI-JP cohort confirmed elevated KIF11 in HCC and an AUC of 93.7%; patients with low KIF11 had a significantly longer OS (HR = 3.745; 95% CI: 1.843–7.612; *p* < 0.001) (Figure 3G–I).

Together, these data demonstrate that KIF11 is robustly overexpressed in HCC and serves as a promising diagnostic and prognostic biomarker.

### 3.2. KIF11 Correlates with Immune Infiltration and PI3K/Akt Pathway Activation

**Figure 4:**
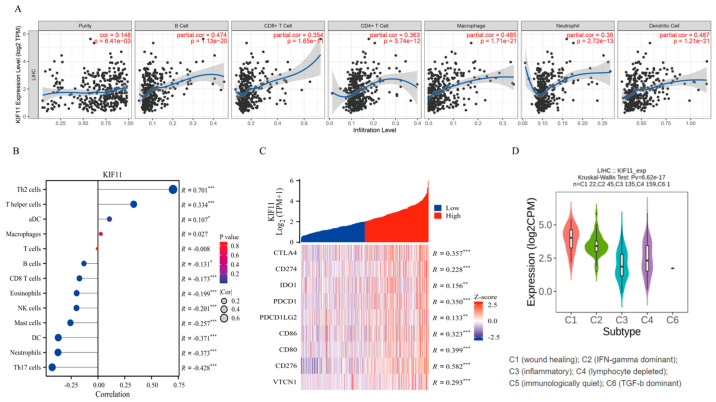
KIF11 and the tumor immune microenvironment in HCC. (**A**) Correlation analysis between KIF11 expression and immune cell infiltration levels, including B cells, CD8^+^ T cells, CD4^+^ T cells, macrophages, neutrophils, and dendritic cells, based on the TIMER2.0 database, using Spearman test. (**B**) Validation of the correlation between KIF11 expression and multiple immune cell types in the TCGA-LIHC cohort, using Spearman test. (**C**) Correlation between KIF11 expression and key immune checkpoint genes in the TCGA-LIHC cohort, using Spearman test. (**D**) Differential expression of KIF11 across molecular immune subtypes in HCC as analyzed by the TISIDB database, using One-way ANOVA. * *p* < 0.05; ** *p* < 0.01; *** *p* < 0.001.

To explore possible mechanisms linking KIF11 to HCC progression, immune and pathway analyses were performed. KIF11 expression correlated significantly with infiltration levels of multiple immune cell types, including B cells, CD8^+^ T cells, CD4^+^ T cells, macrophages, neutrophils and dendritic cells (Figure 4A,B). KIF11 was also positively associated with immune checkpoint genes such as CTLA4, CD274 (PD-L1), and CD86 (Figure 4C), and varied across immune subtypes in TISIDB (Figure 4D), implying immune-related roles.

**Figure 5:**
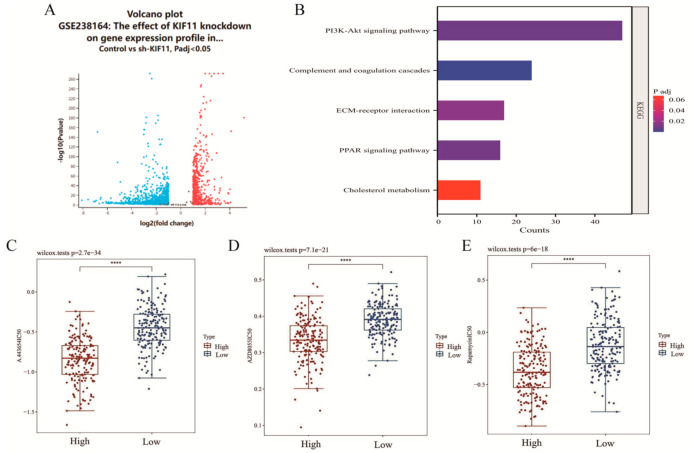
Downstream pathway analysis of KIF11 in HCC. (**A**) Volcano plot visualizing DEGs in the GSE238164 dataset. (**B**) KEGG pathway enrichment analysis of DEGs from GSE238164, highlighting significantly enriched signaling pathways. (**C**–**E**) Comparison of IC50 values for A-443654, AZD8055, and Rapamycin between high- and low-KIF11 expression groups in the GDSC database, illustrating potential therapeutic sensitivities, with Spearman test. **** *p* < 0.0001.

Transcriptomic analysis of GSE238164 (KIF11 knockdown in HepG2) identified differentially expressed genes enriched in the PI3K/Akt signaling pathway by KEGG (Figure 5A,B). Drug sensitivity analysis (GDSC) showed that IC_50_ values for PI3K/Akt pathway inhibitors (A-443654, AZD8055, and Rapamycin) were negatively correlated with KIF11 expression in TCGA-LIHC (*p* < 0.05), suggesting that aberrant KIF11 is associated with altered sensitivity to PI3K/Akt inhibition (Figure 5C–E).

These combined results indicate that KIF11 may drive tumor progression both via modulation of the tumor immune microenvironment and by activating the PI3K/Akt signaling axis.

### 3.3. DHA Inhibits Malignant Phenotypes of HCC Cells In Vitro

**Figure 6:**
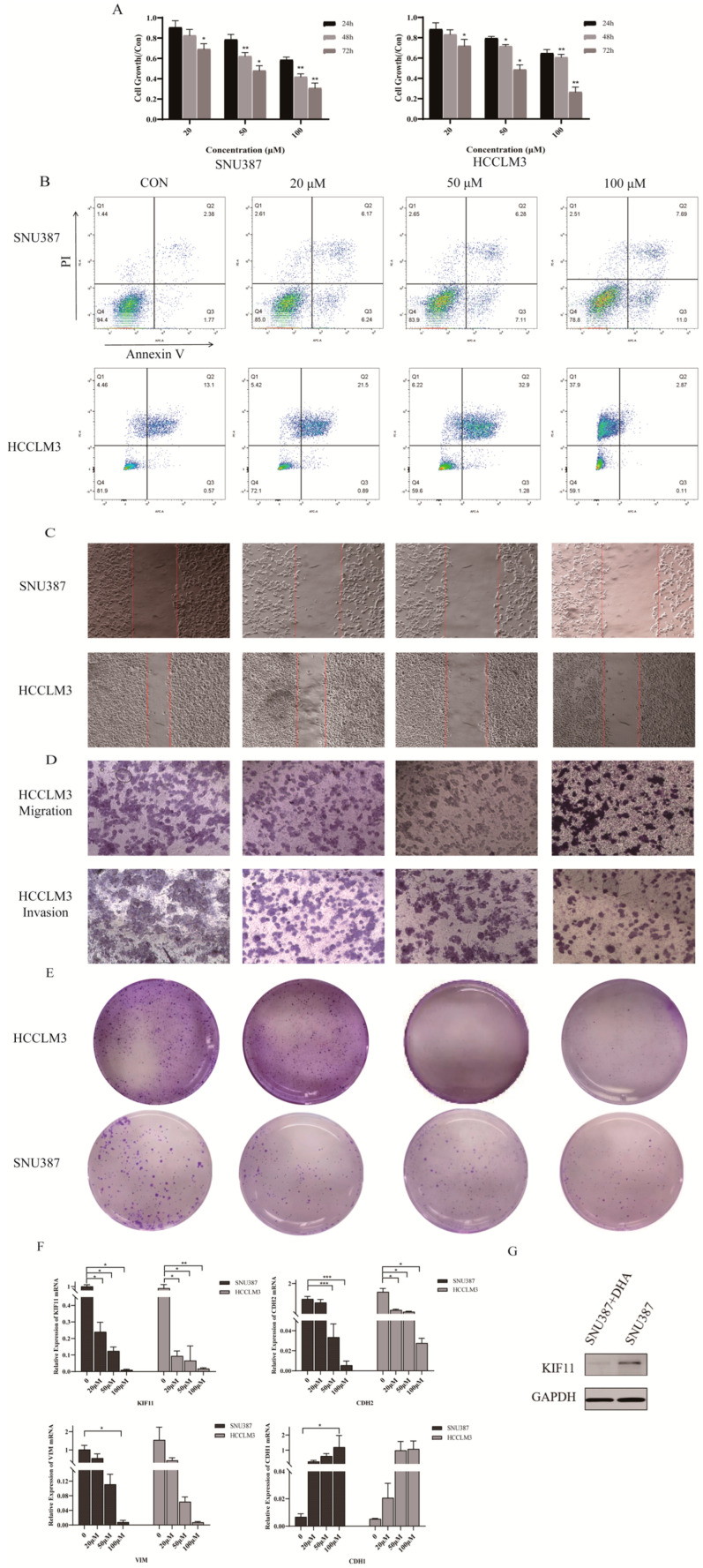
DHA suppresses malignant phenotypes in HCC cells. (**A**) CCK-8 assay evaluating DHA-mediated suppression of HCCLM3 and SNU387 cell proliferation, using a One-way ANOVA with Bonferroni correction. (**B**) FC analysis of DHA-induced apoptosis in HCCLM3 and SNU387 cells, using a One-way ANOVA with Bonferroni correction. (**C**) Wound healing assay showing the reduced migration of HCCLM3 and SNU387 cells upon DHA treatment (scale bar = 50 µm; 100×), using a One-way ANOVA with Bonferroni correction. (**D**) Transwell assay demonstrating DHA-induced inhibition of invasion and migration in HCCLM3 cells (scale bar = 50 µm; 100×), using a One-way ANOVA with Bonferroni correction. (**E**) Colony formation assay quantifying the suppressive effect of DHA on clonogenic potential in HCCLM3 and SNU387 cells, using a One-way ANOVA with Bonferroni correction. (**F**) qRT-PCR analysis showing the downregulation of KIF11, CDH2, and VIM expression in HCCLM3 and SNU387 cells following DHA treatment, along with the upregulation of CDH1, using a One-way ANOVA with Bonferroni correction. (**G**) WB confirming the downregulation of KIF11 protein expression in SNU387 cells upon DHA exposure, with Student’s *t* test. The uncropped blots and molecular weight markers are shown in Supplementary File S1. * *p* < 0.05; ** *p* < 0.01; *** *p* < 0.001.

We next tested whether DHA affects HCC malignant behaviors and KIF11 expression. CCK-8 assays showed that DHA significantly reduced the viability of HCCLM3 and SNU387 cells in a time- and dose-dependent manner (*p* < 0.05). Flow cytometry revealed dose-dependent increases in apoptosis after DHA treatment (*p* < 0.05). Wound-healing and Transwell assays demonstrated markedly impaired migration and invasion in DHA-treated groups (as previously described results of SNU387 in our study [17] (*p* < 0.05), and colony formation was substantially suppressed (*p* < 0.05) (Figure 6A–E).

At the molecular level, qRT-PCR indicated dose-dependent decreases in KIF11, VIM and CDH2 mRNA and an increase in CDH1 (*p* < 0.05). Western blotting confirmed that KIF11 protein was significantly reduced in SNU387 after 24 h treatment with 50 μM DHA (*p* < 0.05) (Figure 6F,G). Collectively, these data show that DHA suppresses proliferation, migration, invasion and EMT-like changes in HCC cells while downregulating KIF11.

### 3.4. KIF11 Overexpression Reverses the Inhibitory Effect of DHA via PI3K/Akt Signaling

**Figure 7:**
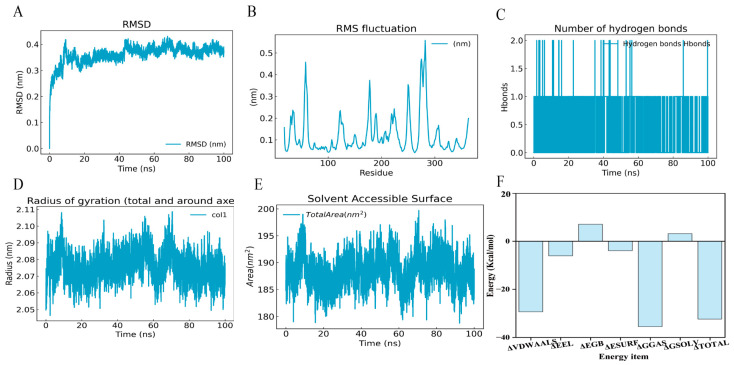
Molecular dynamics stimulation of DHA-KIF11. (**A**) Root-mean-square deviation (RMSD) of the protein–ligand complex. (**B**) Root-mean-square fluctuation (RMSF) of the protein backbone atoms. (**C**) Time evolution of the number of intermolecular hydrogen bonds formed between the protein and ligand. (**D**) Radius of gyration (Rg) of the complex. (**E**) Solvent-accessible surface area (SASA) of the complex. (**F**) Binding-free energy estimates derived from MM/PBSA calculations.

Molecular dynamics simulations were performed to characterize the key amino acid residues mediating the interaction between DHA and KIF11. The complex showed excellent convergence over 100 ns. The RMSD equilibrated after 40 ns, stabilizing at approximately 0.35–0.40 nm, indicating a reliable equilibrium state for analysis (Figure 7A). The radius of gyration (Rg) and SASA remained consistent (averaging ~2.08 nm and ~185 nm^2^ respectively, Figure 7D,E), confirming that the protein maintained a compact, globular fold without significant structural distortion upon ligand binding. Local flexibility analysis (RMSF) identified high stability in the core regions, with increased fluctuations (up to 0.5 nm) localized only in specific loop regions (e.g., residues 270–280). The MM/PBSA analysis (total binding energy) reveals that the association is energetically favorable (Figure 7F). The binding is primarily driven by van der Waals interactions and electrostatic contributions, which outweigh the desolvation penalty (polar solvation energy).

**Figure 8:**
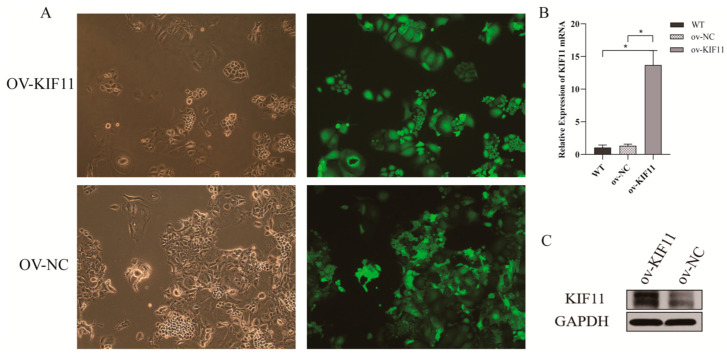
Validation of ov-KIF11 SNU387 cell line. (**A**) Fluorescence microscopy images showing transfection efficiency in ov-KIF11 and ov-NC SNU387 cells (scale bar = 50 µm; 100×). (**B**) qRT-PCR analysis confirming elevated KIF11 mRNA expression in ov-KIF11 cells compared to control, using a One-way ANOVA with Bonferroni correction. (**C**) WB verifying increased KIF11 protein levels, with Student’s *t* test. The uncropped blots and molecular weight markers are shown in Supplementary File S1. * *p* < 0.05.

**Figure 9:**
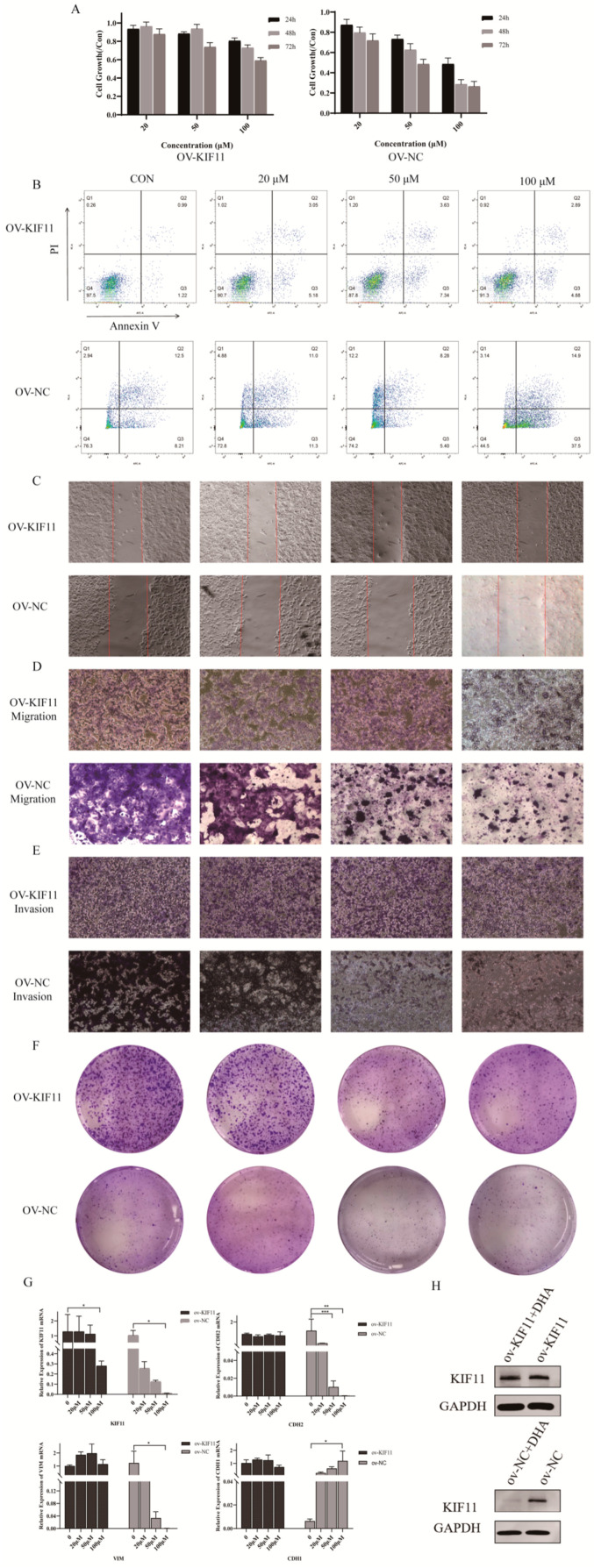
Overexpression of KIF11 attenuates the inhibitory effects of DHA on malignant phenotypes. (**A**) CCK-8 assay assessing DHA-mediated suppression of cell proliferation in ov-KIF11 and ov-NC SNU387 cells, using a One-way ANOVA with Bonferroni correction. (**B**) FC analysis of DHA-induced apoptosis in ov-KIF11 versus ov-NC group, using a One-way ANOVA with Bonferroni correction. (**C**) Wound healing assay showing reduced migration following DHA treatment (scale bar = 50 µm; 100×). (**D**) Transwell assay demonstrating DHA-induced inhibition of cell invasion in ov-NC and ov-KIF11 cells (scale bar = 50 µm; 100×), using a One-way ANOVA with Bonferroni correction. (**E**) Transwell assay quantifying DHA-mediated suppression of cell migration, using a One-way ANOVA with Bonferroni correction. (**F**) Colony formation assay evaluating the clonogenic potential of ov-KIF11 and ov-NC cells upon DHA exposure, using a One-way ANOVA with Bonferroni correction. (**G**) qRT-PCR analysis of KIF11, CDH1, CDH2, and VIM expression levels after DHA treatment, using a One-way ANOVA with Bonferroni correction. (**H**) WB confirming sustained KIF11 protein expression in ov-KIF11 cells despite DHA treatment, with Student’s *t* test.The uncropped blots and molecular weight markers are shown in Supplementary File S1. * *p* < 0.05; ** *p* < 0.01; and *** *p* < 0.001.

To assess whether KIF11 mediates DHA’s effects, a stable ov-KIF11 SNU387 cell line was generated by lentiviral transduction; successful overexpression was confirmed by GFP imaging, qRT-PCR and WB (*p* < 0.05) (Figure 8A–C). Functional assays showed that KIF11 overexpression largely abolished DHA’s inhibitory effects on proliferation, apoptosis induction, migration, invasion and colony formation compared with ov-NC cells (*p* > 0.05) (Figure 9A–F). Correspondingly, qRT-PCR and WB indicated that KIF11, VIM, CDH2 and CDH1 levels remained unchanged after DHA exposure in ov-KIF11 cells (*p* > 0.05) (Figure 9G,H).

**Figure 10:**
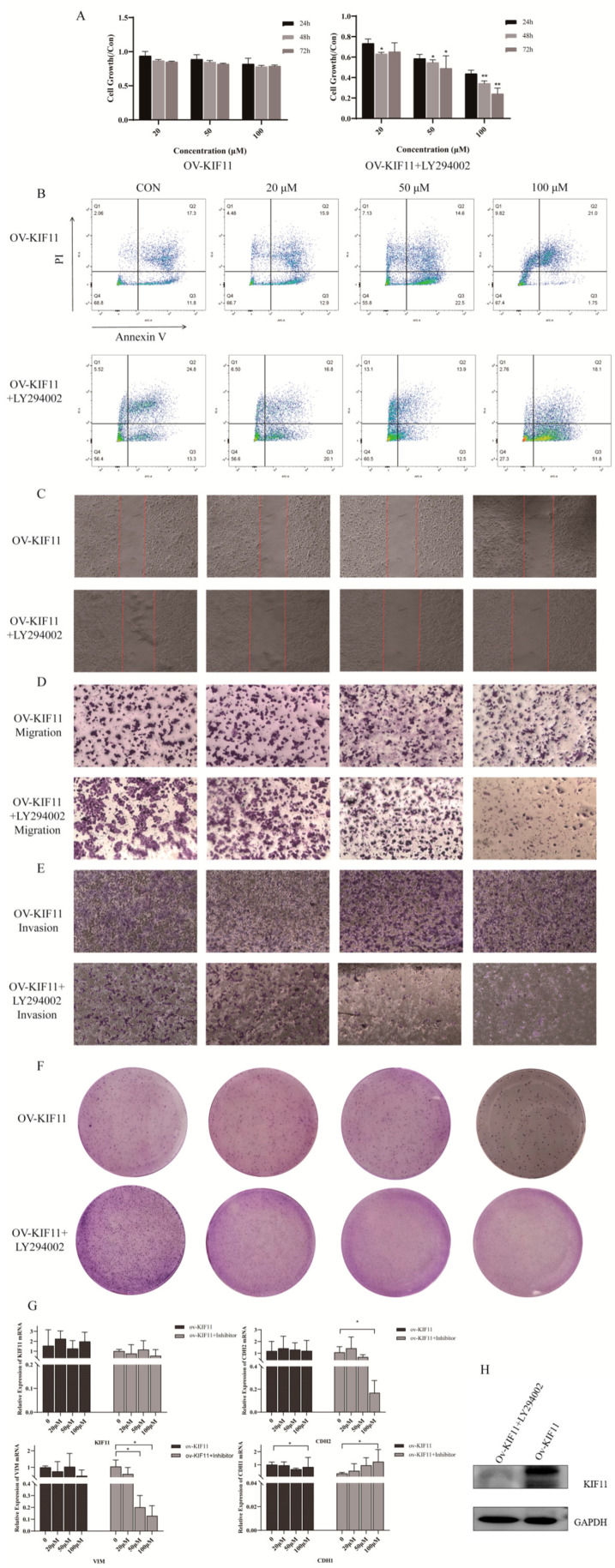
DHA restores suppression on malignant phenotypes of ov-KIF11 cells upon PI3K inhibition. (**A**) CCK-8 assay showing that combined treatment with DHA and LY294002 inhibits proliferation of ov-KIF11 SNU387 cells, using a One-way ANOVA with Bonferroni correction. (**B**) FC analysis demonstrating enhanced apoptosis induction by the combination of DHA and LY294002 in ov-KIF11 SNU387 cells, using a One-way ANOVA with Bonferroni correction. (**C**) Wound healing assay indicating that co-treatment with DHA and LY294002 suppresses cell migration (scale bar = 50 µm; 100×), using a One-way ANOVA with Bonferroni correction. (**D**) Transwell invasion assay revealing reduced invasive capacity of ov-KIF11 SNU387 cells following DHA and LY294002 treatment (scale bar = 50 µm; 100×), using a One-way ANOVA with Bonferroni correction. (**E**) Transwell migration assay confirming inhibition of cell motility by the DHA and LY294002 combination (scale bar = 50 µm; 100×), using a One-way ANOVA with Bonferroni correction. (**F**) Colony formation assay demonstrating significant reduction in colony-forming ability upon co-treatment, using a One-way ANOVA with Bonferroni correction. (**G**) qRT-PCR results showing that the combination treatment does not alter KIF11 expression, but upregulates CDH1 and downregulates CDH2 and VIM, using a One-way ANOVA with Bonferroni correction. (**H**) WB of ov-KIF11 SNU387 cells showing unchanged KIF11 levels with DHA and LY294002, with Student’s *t* test. The uncropped blots and molecular weight markers are shown in Supplementary File S1. * *p* < 0.05; ** *p* < 0.01.

Given the PI3K/Akt link from bioinformatics, we next combined DHA with the PI3K inhibitor LY294002. Co-treatment in ov-KIF11 cells restored sensitivity to DHA, significantly inhibiting proliferation and migration, and increasing apoptosis (*p* < 0.05). Molecular assays showed that while KIF11 expression itself remained unaltered, VIM and CDH2 decreased and CDH1 increased in a drug concentration-dependent manner (*p* < 0.05); WB results were consistent (Figure 10). These rescue experiments indicate that DHA suppresses HCC malignant phenotypes through a KIF11/PI3K/Akt-dependent mechanism.

### 3.5. DHA Suppresses TUMOR Growth and Induces Apoptosis In Vivo

**Figure 11:**
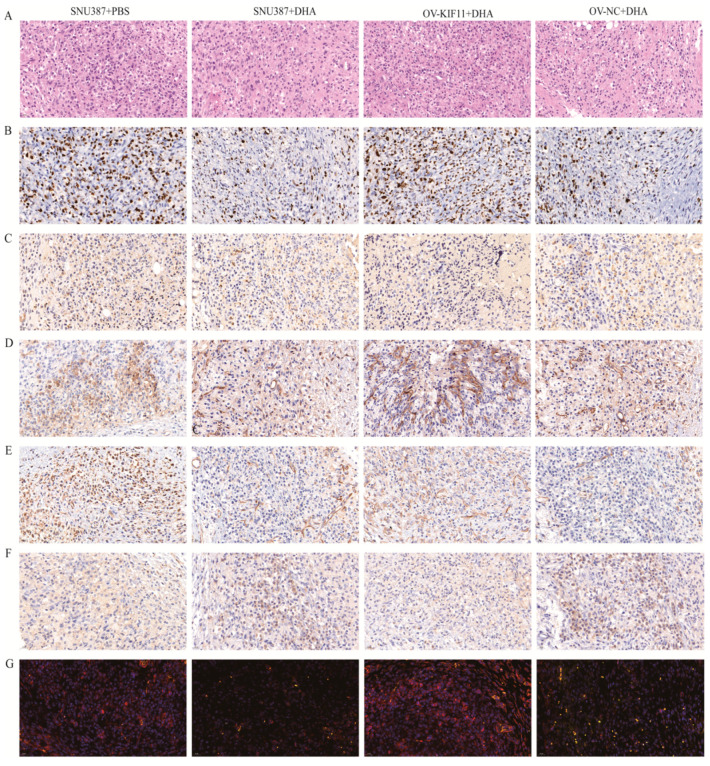
DHA inhibits the growth and progression of HCC in vivo. (**A**) H&E staining of xenograft tumor tissues, revealing reduced malignancy and altered tissue architecture in DHA-treated groups. (**B**) IHC staining for Ki-67 in tumor tissues, indicating proliferative activity, using a One-way ANOVA with Bonferroni correction. (**C**) TUNEL assay to detect apoptotic cells in tumor tissues, showing increased apoptosis in DHA-treated groups, using a One-way ANOVA with Bonferroni correction. (**D**–**F**) IHC detection of CDH2, VIM and CDH1 in tumor tissues, showing reduced expression in DHA-treated groups, using a One-way ANOVA with Bonferroni correction. (**G**) IF staining of KIF11 in tumor tissues (scale bar = 20 µm; 200×).

To further validate our in vitro findings, we established a subcutaneous xenograft model using SNU387 cells. After two weeks of treatment with DHA (50 mg/kg), H&E staining showed that HCC cells in the control group displayed prominent mitotic figures and nuclear atypia, whereas the DHA-treated tumors exhibited lighter nuclear staining and reduced atypia. Tumors derived from ov-KIF11 cells showed a more disorganized cellular architecture and remained morphologically malignant after DHA treatment, suggesting that KIF11 overexpression diminished the antitumor effect of DHA (*p* < 0.05) (Figure 11A). Consistent with these observations, IHC and TUNEL assays revealed a significantly lower proportion of Ki-67-positive cells and a higher proportion of TUNEL-positive apoptotic cells in the DHA-treated tumors compared with controls (*p* < 0.05). However, the ov-KIF11 group exhibited markedly higher Ki-67 and lower TUNEL rates than the ov-NC group under the same treatment conditions (*p* < 0.05) (Figure 11B,C). At the molecular level, VIM and CDH2 expression were significantly decreased, while CDH1 was upregulated in DHA-treated tumor tissues (*p* < 0.05). These EMT-related changes were largely abolished in the ov-KIF11 tumors (Figure 11D–F). Similarly, KIF11 expression in tumor tissues was markedly reduced by DHA, whereas its level remained significantly higher in the ov-KIF11 group even after DHA treatment (*p* < 0.05) (Figure 11G). Collectively, these in vivo results confirm that DHA inhibits tumor growth, suppresses proliferation and EMT, and promotes apoptosis in HCC through a KIF11-dependent mechanism.

## 4. Discussion

In this study, we demonstrated that DHA suppresses proliferation, migration, and invasion while promoting apoptosis in HCC via the KIF11/PI3K/Akt signaling pathway, both in vitro and in vivo. Our data identify KIF11 as a novel molecular effector of DHA and highlight its potential as a diagnostic and prognostic biomarker in HCC. These findings provide mechanistic insight into how a clinically safe, natural compound can selectively inhibit HCC progression. By linking targeted molecular inhibition with phenotypic outcomes, our work offers a promising therapeutic strategy for HCC and underscores the translational potential of DHA in liver cancer therapy.

Artemisinin, a traditional and well-established antimalarial agent derived from Chinese medicine, has a long history of clinical use. The renowned Chinese pharmacologist Tu Youyou was awarded the 2015 Nobel Prize in Physiology or Medicine for her pioneering work in the development and application of artemisinin-based therapies, which significantly advanced global public health. Tu and her team successfully synthesized DHA, the first-generation artemisinin derivative. The hydroxyl group in DHA not only enhances its antimalarial potency but also serves as a structural core for other derivatives, including artesunate, artemether, and arteether [7]. Notably, it has been shown that all artemisinin-based drugs are ultimately metabolized into DHA to exert their therapeutic effects [53]. Studies have demonstrated the promising therapeutic potential of DHA in various conditions, including autoimmune disorders, infectious diseases, inflammatory pathologies, and fibrotic diseases, with growing interest in its anticancer applications. Despite the high prevalence and substantial mortality burden of cancer, clinical oncology faces significant limitations due to severe toxicities and drug resistance associated with conventional chemotherapy and radiotherapy regimens. Our previous study indicated that DHA may emerge as a compelling candidate for cancer therapy owing to its highly selective cytotoxicity toward tumor cells and multi-targeted intervention mechanisms [4].

Our research has identified potential hub genes involved in DHA’s targeting of HCC through network pharmacology [17]. Among all the proteins, KIF11 was selected based on its clinical translational potential and pan-cancer abnormalities. Given the extensive anti-cancer effects of DHA, it represents compelling evidence for its application in the treatment of malignant diseases.

By systematically analyzing transcriptomic sequencing data from multiple repositories and validating the findings experimentally, we demonstrated that KIF11 exhibits pan-cancer overexpression, realizing its potential as a novel diagnostic biomarker in HCC, with significantly elevated levels observed in both HCC cell lines and clinical specimens—consistent with previous reports [54]. Furthermore, KIF11 expression was found to vary across immune subtypes and showed significant correlations with key immune infiltrates, including CD8^+^ T cells, CD4^+^ T cells, and neutrophils, as well as immune checkpoint genes such as CTLA4 and CD274. Based on the GSE238164 dataset entitled “The effect of KIF11 knockdown on gene expression profile in HepG2 cells,” KEGG enrichment analysis revealed that the majority of DEGs are enriched in the PI3K/Akt pathway, suggesting that KIF11 exerts its oncogenic functions in HCC through modulation of the PI3K/Akt signaling axis. Moreover, the association between KIF11 and the PI3K/Akt pathway is well-established [55,56,57]. Accumulating evidence indicates that DHA modulates the PI3K/Akt signaling pathway across various tumor types. Rao et al. demonstrated that DHA inhibits the PI3K/Akt/NF-κB pathway in MDA-MB-231 breast cancer cells [58]. Wu et al. reported that DHA downregulates the PI3K/Akt pathway in HCCLM6 cells [59]. Fu et al. confirmed in cellular experiments that DHA suppresses angiogenesis in glioma stem cells via the Epha2/PI3K/Akt signaling axis [60]. Moreover, the assessment of key downstream effectors—including phosphorylated Akt (Ser473 and Thr308), mTOR, GSK3β, and cleaved caspase-3/9—would provide a more comprehensive and mechanistically grounded understanding of the signaling cascade. Notably, prior studies have reported that KIF11 expression is modulated by GSK3β activity [61], caspase-3/7 activation [62], and Akt phosphorylation status [55], suggesting its functional integration possibly. Collectively, we hypothesize that DHA exerts its therapeutic effects in HCC by acting via KIF11/PI3K/Akt signaling pathway, with more mechanism details to be explored.

To investigate the effect of KIF11 overexpression on the proliferation, apoptosis, migration, and invasion of HCC cells, stable KIF11-overexpressing (ov-KIF11) SNU387 cells were successfully established via lentiviral infection. Phenotypic assays demonstrated that the inhibitory effect of DHA on ov-KIF11 cells was significantly attenuated. Notably, the suppressive effect of DHA was restored upon treatment with the PI3K inhibitor LY294002. RT-PCR and Western blot analyses showed no significant change in KIF11 expression levels, suggesting that KIF11 functions as a role of upstream regulator of the PI3K/Akt signaling pathway. A nude mouse xenograft model was established using both ov-KIF11 and control SNU387 cells, with intraperitoneal administration of DHA to validate the in vitro findings. DHA treatment markedly suppressed tumor growth and malignancy, as evidenced by reduced Ki-67 and VIM expression, increased TUNEL staining, and modulation of CDH1 and CDH2 levels; however, these therapeutic effects were partially abrogated in the ov-KIF11 group. Immunofluorescence staining confirmed downregulation of KIF11 following DHA treatment. Collectively, these results demonstrate that KIF11 overexpression counteracts the antitumor effects of DHA in HCC, underscoring its critical role in mediating DHA-induced tumor suppression.

In conclusion, this study demonstrates that DHA suppresses HCC proliferation, migration, and invasion while inducing apoptosis through the KIF11/PI3K/Akt signaling pathway, as was validated in vitro and in vivo. These findings provide a theoretical foundation for the potential clinical application of DHA in liver cancer therapy. Nevertheless, there are limitations that could be improved. (1) KIF11 was prioritized based on integrated analyses using network pharmacology and bioinformatics approaches. However, these computational predictions remain preliminary and inherently subject to methodological limitations—including database biases, algorithmic assumptions, and lack of biological validation. Robust experimental confirmation—such as clinical correlation analysis in well-annotated patient cohorts, functional assays in vitro, and mechanistic studies in vivo—is therefore essential before drawing definitive biological or translational conclusions. A comprehensive validation strategy, encompassing orthogonal experimental modalities and independent clinical cohorts, is planned for subsequent work. (2) Surface plasmon resonance, microscale thermophoresis, and cellular thermal shift assays can be utilized to confirm the direct binding predicted by molecular docking and dynamics stimulation. (3) Orthotopic or immunocompetent mouse models as well as a second HCC line with functional and genetic engineering would strengthen confidence of mechanistic conclusions. (4) In vivo pharmacokinetic/pharmacodynamic (PK/PD) profiling—including systemic exposure, target engagement, and downstream biomarker modulation—along with comprehensive metabolic characterization (e.g., hepatic clearance and major metabolite identification), would strengthen mechanistic interpretation and support translational relevance. (5) Co-immunoprecipitation coupled with mass spectrometry may be applied to determine whether KIF11 directly interacts with components of the PI3K pathway. Further mechanistic exploration will facilitate the rational design and optimization of novel therapeutic agents targeting this axis.

## 5. Conclusions

Previously, we stated that DHA is safe and desirable for clinical application according to ADME/T evaluation. Among hub genes, KIF11 was chosen for further validation for its potency in the occurrence, diagnosis and prognosis of HCC. In this study, we have proven that DHA suppresses HCC by acting on KIF11 and PI3K/Akt modulation comprehensively both in vivo and in vitro. These novel findings offer theoretical basis for drug development and protein modification.

## Data Availability

The datasets used and/or analyzed during the current study are available from the corresponding author on reasonable request.

## Supplementary Materials

The following supporting information can be downloaded at: https://www.mdpi.com/article/10.3390/cancers18101530/s1, Table S1: Primer Sequences; Supplementary File S1: Original Image of Western Blot.
