# Supplementary material for: Dihydroartemisinin Suppresses Hepatocellular Carcinoma Progression by Acting on KIF11 with PI3K/Akt Modulation
Source: Cancers (Basel). 2026 May 9;18(10):1530. doi: 10.3390/cancers18101530 (PMC13204978; doi:10.3390/cancers18101530)
Supplement: Supplementary file 1 [file cancers-18-01530-s001.zip › cancers-4258236-Supplementary Table S1.pdf]

Supplementary Table S1. Primer Sequences

Gene	Species	Sequence	GenBank Accession	Amplicon Size	Primerbank ID
GAPDH-F	Human	GTCTCCTCTGACTTCAACAGCG	NM_001357943.2	131	-
GAPDH-R	Human	ACCACCCTGTTGCTGTAGCCAA			
KIF11-F	Human	TCCCTTGGCTGGTATAATTCCA	NM_004523	181	197304797c1
KIF11-R	Human	GTTACGGGGATCATCAAACATCT			
VIM-F	Human	AATGACCGCTTCGCCAAC	NM_003380.5	135	-
VIM-R	Human	CCGCATCTCCTCCTCGTAG			
CDH1-F	Human	CGAGAGCTACACGTTCACGG	NM_004360	119	169790842c1
CDH1-R	Human	GGGTGTCGAGGGAAAAATAGG			
CDH2-F	Human	TCAGGCGTCTGTAGAGGCTT	NM_001792	94	215422305c1
CDH2-R	Human	ATGCACATCCTTCGATAAGACTG			
