# Supplementary figures and images for: Dihydroartemisinin Suppresses Hepatocellular Carcinoma Progression by Acting on KIF11 with PI3K/Akt Modulation
Source: Cancers (Basel). 2026 May 9;18(10):1530. doi: 10.3390/cancers18101530 (PMC13204978; doi:10.3390/cancers18101530)

Figure 2 c

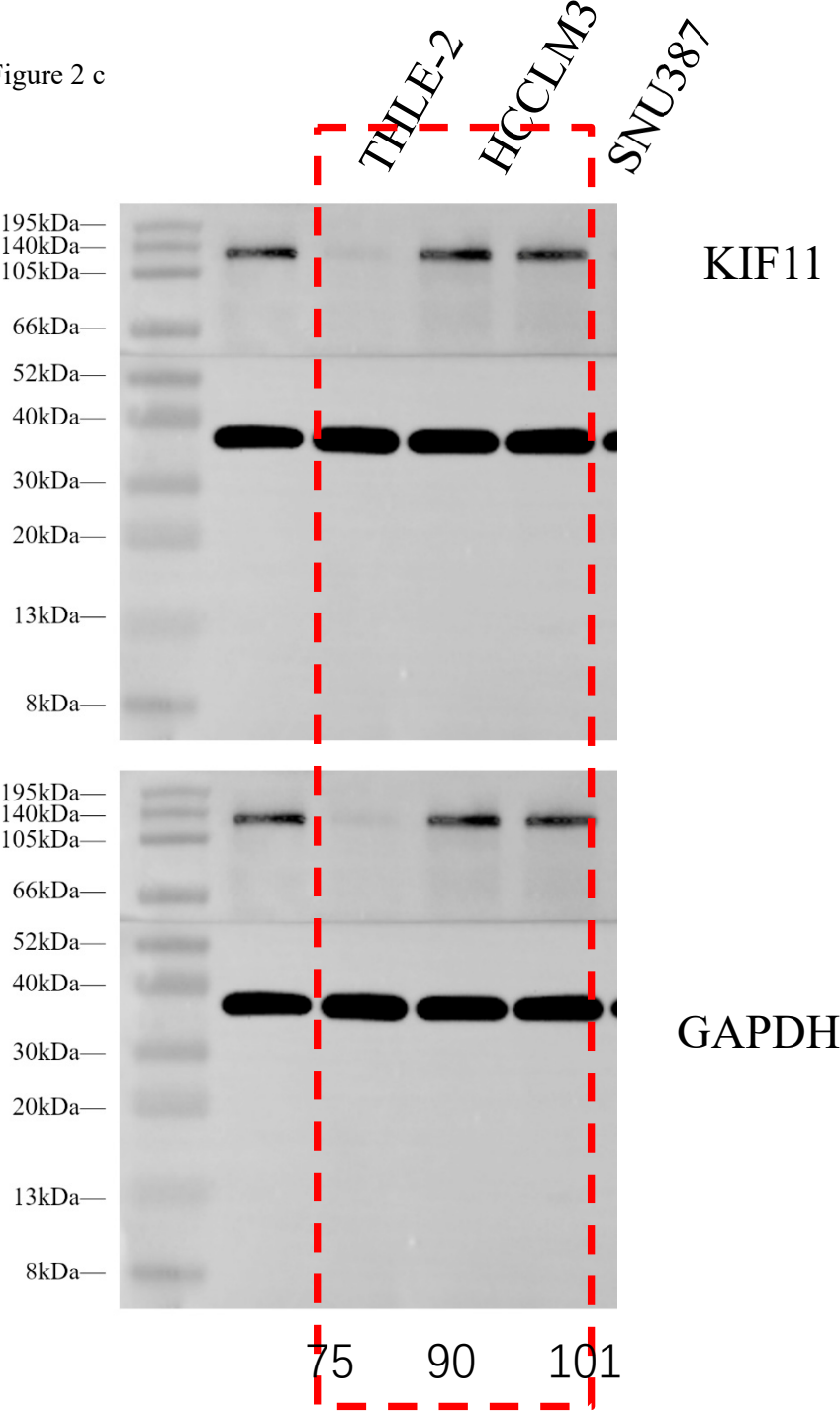

Figure 6 g

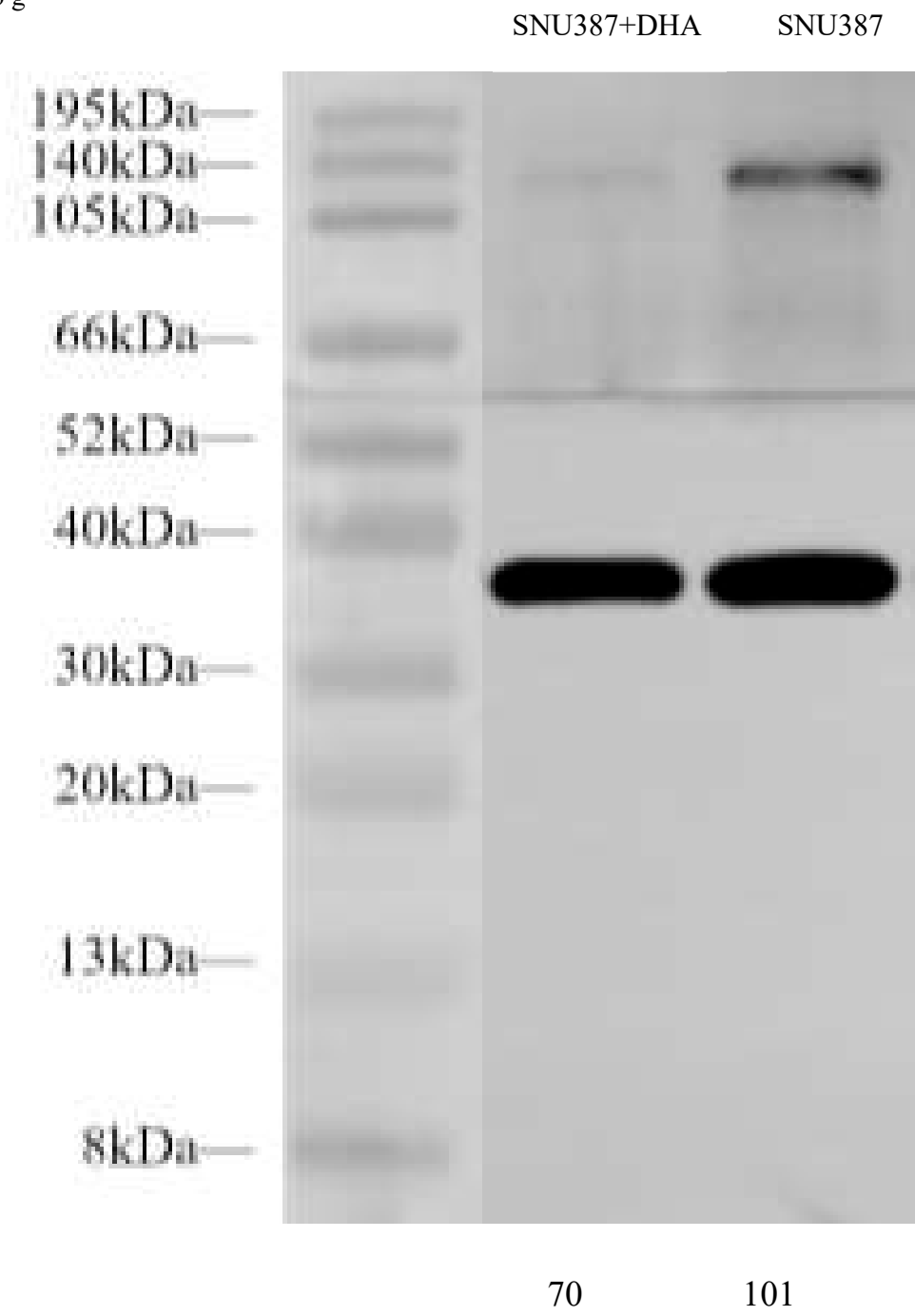

Figure 8 c

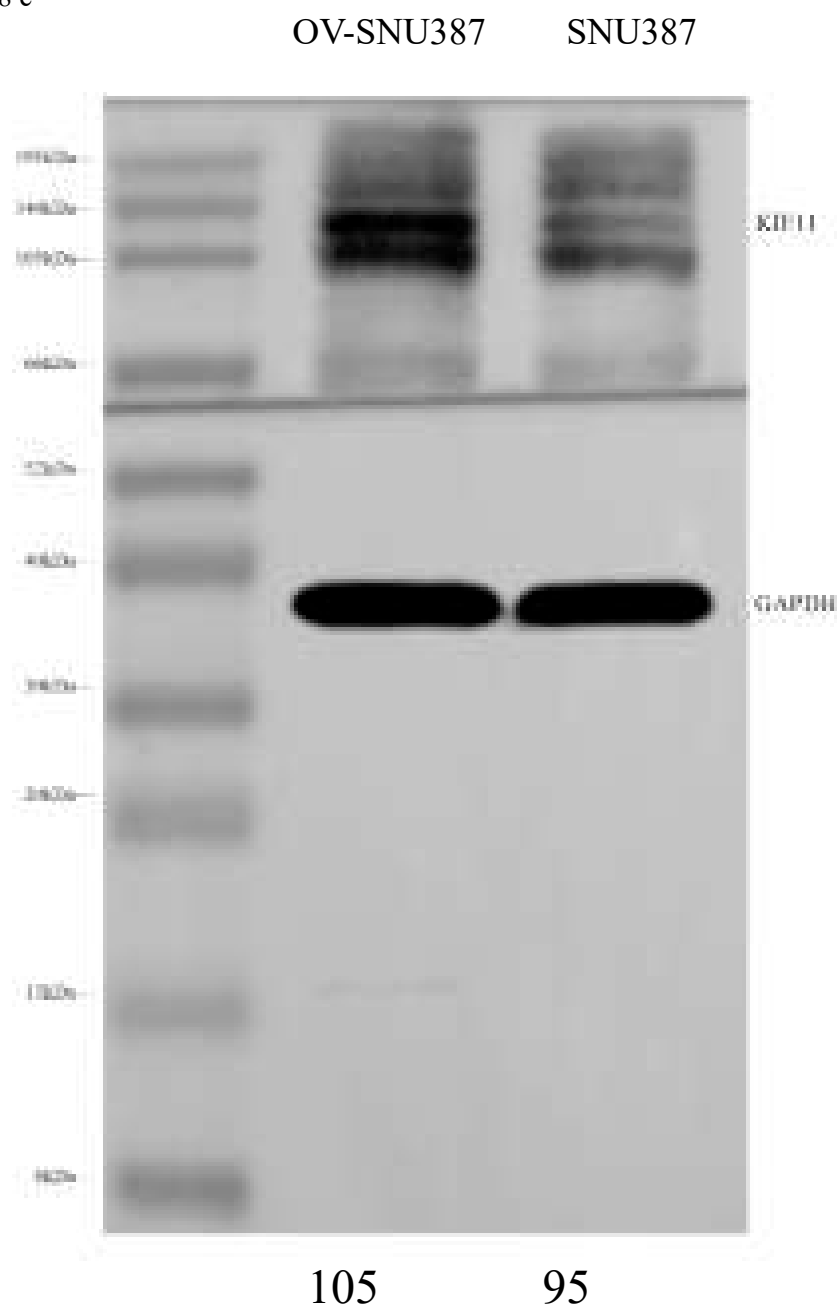

Figure 9 h

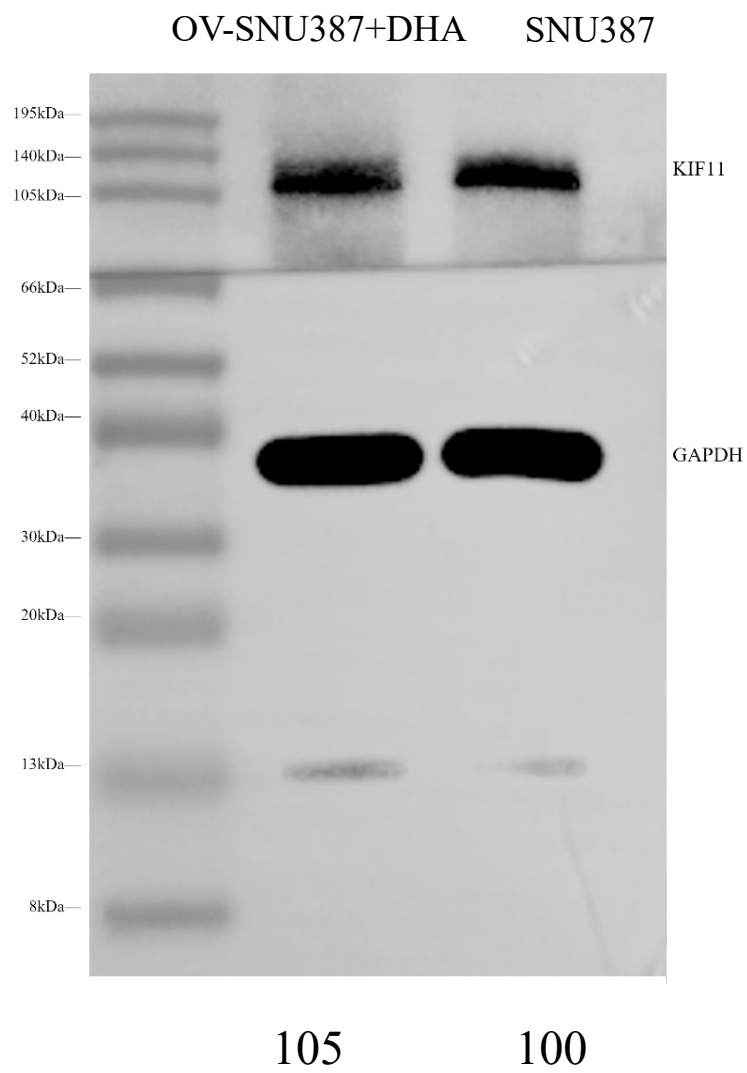

Figure 9 h

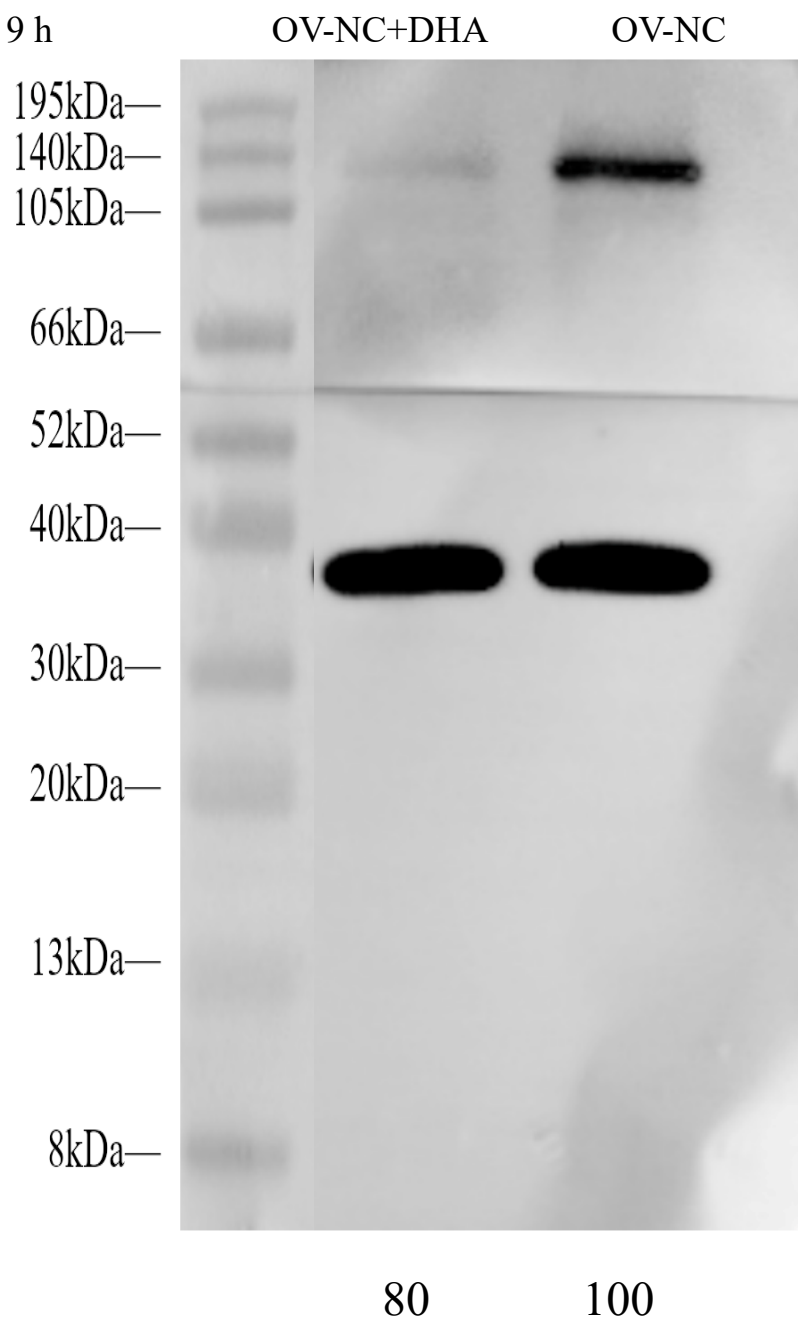

Figure 10 h

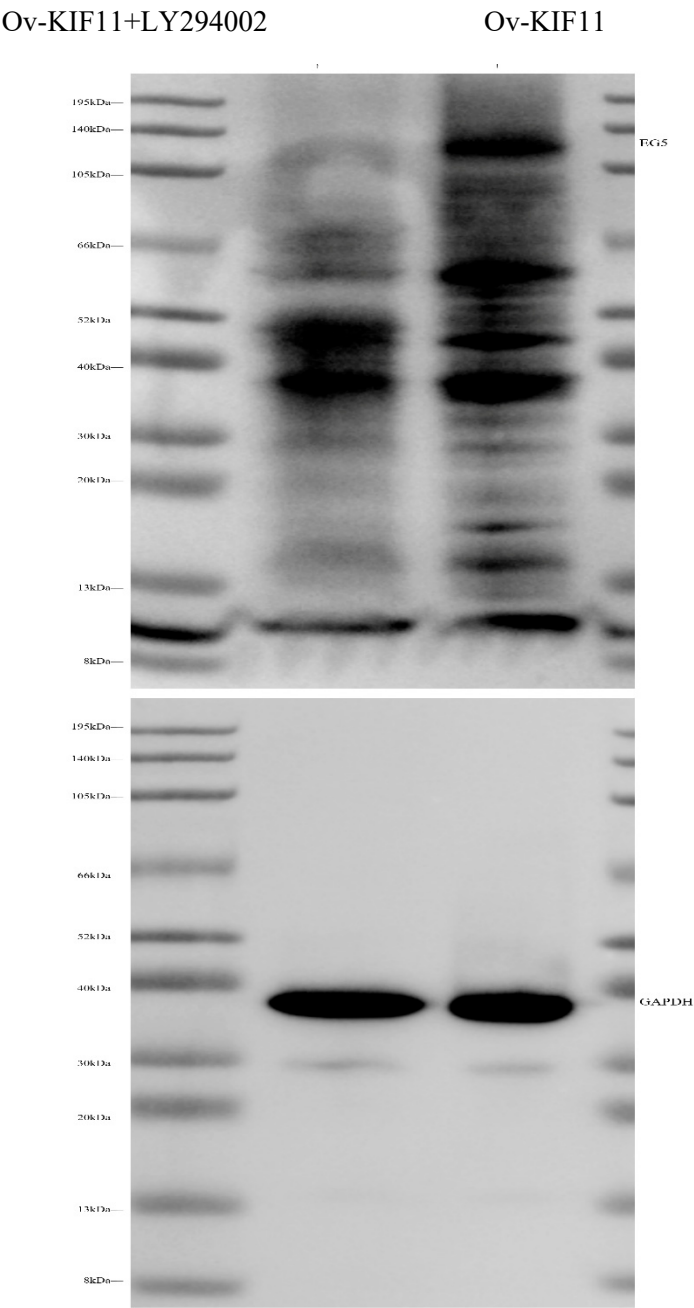

Supplement: Supplementary file 1 [file cancers-18-01530-s001.zip › cancers-4258236-Supplementary File S1.pdf]
